# Psychological and Glycemic Outcomes Associated with EMDR Therapy in Children and Adolescents with Type 1 Diabetes Mellitus: A Retrospective Comparative Study

**DOI:** 10.3390/children13091262

**Published:** 2026-09-17

**Authors:** Sami Arslanoğlu, Mehmet Karadağ, Baran Çalışgan

**Affiliations:** 1 Department of Child and Adolescent Psychiatry, Gaziantep City Hospital, 27470 Gaziantep, Türkiye; 2 Department of Child and Adolescent Psychiatry, Faculty of Medicine, Gaziantep University, 27310 Gaziantep, Türkiye

**Keywords:** EMDR, type 1 diabetes mellitus, anxiety, depression, adverse childhood experiences, children, adolescents, glycemic control

## Abstract

**Highlights:**

**What are the main findings?**

- In children and adolescents with type 1 diabetes, EMDR therapy was associated with significantly greater improvement than standard psychiatric follow-up in separation anxiety, depressive symptoms, and overall anxiety burden, based on significant time × group interactions and within-group comparisons.
- EMDR therapy was also associated with a significantly greater reduction in mean blood glucose than standard psychiatric follow-up, which showed no significant within-group change over the same period; a similar pattern was observed for insulin injection frequency, a secondary, treatment-related measure.

**What are the implications of the main findings?**

- EMDR may be a valuable adjunctive psychological intervention for anxiety and depressive symptoms in youth with type 1 diabetes, with a possible additional association with reduced blood glucose that warrants prospective confirmation.
- Prospective, adequately powered randomized controlled trials with longer follow-up are needed to confirm EMDR’s efficacy and clarify its relationship with metabolic outcomes.

**Abstract:**

Background: Children and adolescents with type 1 diabetes mellitus (T1DM) are at increased risk of comorbid anxiety and depressive symptoms, which can adversely affect glycemic control. Eye Movement Desensitization and Reprocessing (EMDR) therapy has demonstrated efficacy for trauma-related and anxiety symptoms, but its use in pediatric T1DM populations has been rarely studied. Methods: In this retrospective comparative study, 52 children and adolescents with T1DM and comorbid anxiety/depressive symptoms were classified into two equally sized groups according to whether they had received EMDR, based on real-world clinical decision-making rather than randomization: an EMDR group (n = 26) and a comparison group receiving standard psychiatric follow-up without EMDR (n = 26). Anxiety and depressive symptoms were assessed with the Revised Child Anxiety and Depression Scale (RCADS; standardized T-scores) before and after the intervention period. Three-month average blood glucose and mean daily insulin injection frequency were recorded before and after the same period. Group differences were analyzed using two-way repeated-measures analysis of variance (time × group) with Fisher’s LSD post hoc comparisons; baseline continuous and categorical variables were compared using the independent-samples *t*-test and chi-square test, respectively. Results: The EMDR and comparison groups did not differ significantly in age, sex, or other sociodemographic/clinical characteristics (all *p* > 0.05); baseline RCADS scores were comparable between groups except for obsessive–compulsive symptoms, which were higher in the comparison group (62.92 ± 11.24 vs. 55.38 ± 12.76; *p* = 0.020). There was a significant main effect of time (i.e., an overall improvement averaged across both groups) for all eight RCADS subscales (all *p* ≤ 0.02); within-group comparisons showed significant improvement in the EMDR group on every subscale, whereas the comparison group did not improve significantly on depression (*p* = 0.130) or total anxiety-plus-depression (*p* = 0.247). A significant time × group interaction, indicating significantly greater improvement in the EMDR group, was found for separation anxiety (*p* = 0.033), depression (*p* < 0.001), total anxiety (*p* = 0.011), and total anxiety-plus-depression (*p* = 0.015); interactions for generalized anxiety, panic, social phobia, and obsessive–compulsive symptoms did not reach significance (*p* = 0.07–0.86). There was also a significant main effect of time for mean blood glucose and for insulin injection frequency; however, within-group comparisons showed a significant reduction in the EMDR group only, with no significant change in the comparison group for either measure (*p* = 0.719 and *p* = 0.163, respectively). A significant time × group interaction confirmed a substantially greater reduction in the EMDR group for both mean blood glucose (F = 40.45, *p* < 0.001) and insulin injection frequency (F = 19.45, *p* < 0.001). Conclusions: EMDR therapy was associated with greater improvement than standard psychiatric follow-up in separation anxiety, depressive symptoms, and overall anxiety burden, and, notably, with a significantly greater reduction in mean blood glucose; a parallel pattern was observed for insulin injection frequency, a secondary, treatment-related measure that should be interpreted with caution. Given the retrospective, non-randomized design and the post hoc identification of these outcome patterns, these findings should be interpreted as exploratory and hypothesis-generating, warranting confirmation in prospective controlled trials.

## 1. Introduction

Type 1 diabetes mellitus (T1DM) is one of the most common chronic endocrine disorders of childhood and requires lifelong self-management involving insulin administration, glucose monitoring, and dietary regulation. The chronic and demanding nature of this disease places a substantial psychological burden on affected children and their families, and numerous studies have reported elevated rates of anxiety and depressive disorders among youth with T1DM compared with healthy peers [1,2]. Psychological distress in this population is not merely a comorbid concern but is bidirectionally linked with metabolic control: anxiety and depressive symptoms are associated with poorer treatment adherence, higher glycated hemoglobin levels, and an increased risk of acute and chronic diabetes-related complications [3,4].

Adverse Childhood Experiences (ACEs)—including exposure to family conflict, parental separation, bereavement, or maltreatment—have been consistently associated with poorer physical and mental health outcomes across the lifespan, plausibly via cumulative dysregulation of stress-response biology (allostatic load) [5,6], and a growing body of evidence suggests that a higher cumulative ACE burden may compound the psychological and metabolic vulnerability of children living with a chronic illness such as T1DM.

Eye Movement Desensitization and Reprocessing (EMDR) is a structured psychotherapeutic approach originally developed for the treatment of post-traumatic stress disorder, in which bilateral sensory stimulation (visual, tactile, or auditory) is paired with guided processing of distressing memories [7]. Beyond trauma-focused indications, EMDR has increasingly been applied to broader pediatric anxiety and mood symptoms, as illustrated by a randomized comparison of EMDR with cognitive behavioral therapy in disaster-exposed children [8]; however, controlled evidence specifically in children and adolescents with chronic physical illness such as T1DM remains scarce.

Children and adolescents coping with a chronic illness such as T1DM are not simply smaller versions of adult patients: the psychological impact of illness, and the likely benefit of a given psychosocial intervention, may depend on developmental stage, family involvement, cognitive maturity, and the way illness-related distress is experienced and communicated at different ages. Developmentally informed frameworks for psychosocial intervention have been described in other areas of pediatric chronic illness; for example, a recent lifespan-based review of psychosocial interventions in pediatric oncology highlights how developmentally targeted approaches can support emotional regulation and psychosocial adjustment in children and adolescents managing serious medical conditions [9]. Although this literature originates outside the diabetes field, it offers a broader developmental rationale for psychosocial intervention in pediatric chronic illness that complements the more disease-specific T1DM literature cited above.

The aim of the present retrospective study was therefore twofold: first, to compare changes in anxiety and depressive symptoms, assessed with the Revised Child Anxiety and Depression Scale (RCADS), between children with T1DM who received EMDR therapy and those who received standard psychiatric follow-up alone; and second, to explore whether these psychological changes were paralleled by improvements in glycemic parameters (mean blood glucose and insulin injection frequency), and whether cumulative adverse childhood experiences were associated with baseline psychological or metabolic status.

## 2. Materials and Methods

### 2.1. Study Design and Participants

This retrospective comparative study, reported in accordance with the STROBE guidelines for observational studies [10], was conducted using the clinical records of children and adolescents aged 8–16 years who were followed for T1DM (minimum disease duration of 1 year) and were referred to child and adolescent psychiatry for comorbid anxiety and/or depressive symptoms; the presence of these symptoms was established through clinical interview together with RCADS-CV scale scores. Data were extracted from the hospital information system and psychiatric outpatient files. The 26 patients in the EMDR group had been referred to psychiatry because of an additional (comorbid) psychiatric illness and subsequently received a course of EMDR therapy in addition to routine psychiatric follow-up; trauma exposure or PTSD-related symptoms were not specifically used as a criterion for this referral. The 26 patients in the comparison group were randomly selected from among eligible patients who had not received EMDR and received routine psychiatric follow-up alone, reflecting real-world clinical decision-making rather than randomized allocation to treatment. Inclusion was restricted to patients with a stable treatment regimen throughout the observation period: patients whose psychiatric medication regimen or T1DM treatment regimen was changed during follow-up were not included, although dose increases within the same regimen were permitted. Exclusion criteria also included the presence of intellectual disability, a comorbid psychotic disorder, or incomplete pre- or post-intervention records.

Sociodemographic and clinical variables extracted included age, sex, education level, parental age, parental education and occupation, parental marital status, personal and parental psychiatric history, and current use of psychiatric medication.

### 2.2. EMDR Intervention

Patients in the EMDR group received a standardized course of EMDR therapy delivered by a trained child and adolescent psychiatrist, following the eight-phase EMDR protocol adapted for children. The treating psychiatrist had completed the EMDR International Association (EMDRIA)-approved training course “Integrating EMDR into Your Clinical Practice” (EMDR Consulting; 20 h of lecture, 20 h of practicum, and 10 h of consultation), completed in Istanbul, Turkey, on 11 March 2023 under trainer Roy Kiessling, LISW. Patients received a mean of 3 EMDR sessions (range 2–5), each lasting a standard 45 min, delivered weekly over a total treatment period of approximately 3–4 weeks. Only patients who completed the planned course of sessions were included in the study. Therapeutic targets were individualized according to each patient’s identified sources of anxiety or low mood; diabetes-related distress was not used as a specific or predefined EMDR target. Protocol adherence was not evaluated using a standardized fidelity checklist, which is noted as a limitation (Section 4). As described in Section 2.1, psychiatric medication and T1DM treatment regimens were required to remain stable (aside from dose increases) throughout the observation period for both groups. Bilateral stimulation was delivered using one of three modalities according to patient preference and clinical suitability: visual (eye movement), tactile, or auditory stimulation, with no pre-specified algorithm governing this choice. Because only 9, 9, and 7 EMDR-group participants received visual, tactile, and auditory stimulation, respectively (modality data missing for 1 patient), this study was not powered to examine whether treatment modality influenced outcomes, and this heterogeneity should be considered when interpreting the EMDR group’s results (see Section 4). Follow-up (“control”) data were obtained approximately 3 months after baseline in both groups, consistent with the 3-month pre- and post-intervention windows used for the glycemic parameters (Section 2.3). The comparison group continued routine psychiatric follow-up (clinical interviews and, where indicated, pharmacotherapy) without EMDR during the same observation period. The typical frequency and content of these follow-up visits, and the proportion of comparison-group patients who received pharmacotherapy and with which agent classes, were not systematically documented in these retrospective records and could not be reported in further detail; this is acknowledged as a limitation (Section 4).

### 2.3. Measures

Anxiety and depressive symptoms were assessed using the child self-report version of the Revised Child Anxiety and Depression Scale (RCADS-CV), a validated screening instrument comprising subscales for separation anxiety, generalized anxiety, panic disorder, social phobia, obsessive–compulsive symptoms, and major depressive disorder, in addition to composite total anxiety and total anxiety-plus-depression scores [11]. Raw subscale and composite scores were converted to standardized T-scores (population mean = 50, SD = 10) using the normative reference values reported in the original RCADS validation by Chorpita et al. [11] and the Turkish validation by Gormez et al. [12], the latter of which was used in this study. The RCADS-CV was administered at baseline (pre-intervention) and following the intervention period (post-intervention/follow-up) in both groups; as a self-report instrument, the same informant (the child/adolescent) completed both assessments for a given participant. Administration followed the standard self-report format across the full 8–16-year age range; whether individual younger children required assistance reading items was not systematically recorded in these retrospective files, and this is acknowledged as a limitation (Section 4).

Cumulative early-life adversity was quantified using the Negative Life Events Scale (Olumsuz Yaşam Olayları Listesi), a clinician/chart-based checklist of thirteen adverse life-event domains (e.g., witnessing a crime or accident, exposure to violence or maltreatment, serious illness or bereavement of a close friend or family member, parental separation/divorce, and parental legal or incarceration history) reconstructed from clinical records rather than a standardized, prospectively administered ACE questionnaire; no published psychometric reference for this checklist is cited here. Domain-level endorsement rates are reported in Section 3.5. A total score on this checklist and its correlation with baseline psychological and glycemic measures were not computed for this study.

Glycemic control was operationalized as the three-month average blood glucose value, recorded for the three months preceding and the three months following the intervention period, extracted from clinical follow-up records. Blood glucose values were obtained from an implantable continuous glucose monitoring (CGM) sensor, replaced monthly, with readings officially recorded and retrieved through the Republic of Turkey Ministry of Health’s national health information system; device- and manufacturer-specific details are not retrievable from this centralized system, and the exact number of valid sensor readings per patient per three-month window was not available. Whether sensor use and reading availability were fully comparable between groups could not be verified from this system and is acknowledged as a limitation (Section 4). Although the CGM system could in principle provide time-in-range and other detailed glycemic metrics, only the three-month average glucose value was extracted for this study; HbA1c and time-in-range data were not consistently available in these retrospective records and their absence is acknowledged as a limitation (Section 4). Mean daily insulin injection frequency—the actual number of insulin administrations recorded per day, rather than the number prescribed by the treatment regimen—was additionally recorded as a secondary, treatment-related and exploratory measure, separate from blood glucose as the primary glycemic indicator. Because patients with a change in prescribed T1DM treatment regimen during the observation period were not included in the study (Section 2.1), a reduction in injection frequency in this sample is more likely to reflect a change in the actual number of administered doses (e.g., missed or consolidated doses, reflecting adherence) than a formal switch in regimen or insulin pump initiation; injection frequency should therefore not be interpreted as equivalent to, or as direct evidence of, improved glycemic control.

### 2.4. Statistical Analysis

Continuous variables are presented as mean ± standard deviation, and categorical variables as frequency (percentage). Baseline continuous sociodemographic variables were compared between groups using the independent-samples *t*-test, and categorical variables were compared using the chi-square test. Changes in RCADS subscale scores and glycemic parameters (mean blood glucose and insulin injection frequency) over the intervention period were analyzed using two-way repeated-measures analysis of variance, with time (pre- vs. post-intervention) as the within-subjects factor and group (EMDR vs. comparison) as the between-subjects factor; the time × group interaction term was used to test whether the magnitude of change differed between groups, and the main effect of group was used to test overall between-group differences collapsed across time. Fisher’s least significant difference (LSD) test was used for post hoc pairwise comparisons among the four pre-/post-intervention cells (EMDR-pre, EMDR-post, comparison-pre, comparison-post) for each outcome. No outcome was designated as primary in the original study protocol; the eight RCADS subscales, the two glycemic parameters, and the ACE-related analyses are therefore all reported and interpreted as exploratory, without a pre-specified outcome hierarchy. No formal correction for multiple comparisons was applied across these outcome families; given the number of comparisons performed relative to the sample size, results should be interpreted with appropriate caution, and an approach such as a false discovery rate correction could be applied in future work with access to the raw data. Ninety-five percent confidence intervals for the reported effect sizes (partial η^2^) and between-group differences could not be computed from the aggregated SPSS output available for this study and are not reported; this is acknowledged as a limitation (Section 4). A two-tailed *p*-value < 0.05 was considered statistically significant. Analyses were performed using SPSS version 22.0.

### 2.5. Ethical Considerations

This study was approved by the Gaziantep University Clinical Research Ethics Committee (Approval No: 2024/262, approval date: 31 July 2024) and was conducted in accordance with the Declaration of Helsinki [13]. As a retrospective file review of routinely collected clinical data, the requirement for individual informed consent was waived by the ethics committee; all data were anonymized prior to analysis.

## 3. Results

The final sample comprised 52 children and adolescents (26 in the EMDR group and 26 in the comparison group). Mean age was 12.4 ± 2.6 years in the EMDR group and 12.9 ± 2.8 years in the comparison group (*p* = 0.506, independent-samples *t*-test). The proportion of male participants was higher in the EMDR group (57.7%) than in the comparison group (34.6%), but this difference did not reach statistical significance (*p* = 0.095, chi-square test); no other sociodemographic or clinical characteristic differed significantly between groups (Table 1).

### 3.1. Baseline Group Comparison

At baseline, RCADS subscale scores did not differ significantly between groups, with the exception of obsessive–compulsive symptoms, which were significantly higher in the comparison group than in the EMDR group (62.92 ± 11.24 vs. 55.38 ± 12.76; *p* = 0.020, Fisher’s LSD). No other baseline RCADS subscale differed significantly between groups (all *p* ≥ 0.28; Table 2).

### 3.2. Time and Time × Group Effects over the Intervention Period

Two-way repeated-measures ANOVA showed a significant main effect of time (i.e., improvement from pre- to post-intervention) for all eight RCADS subscales (F = 23.98–45.01, all *p* < 0.001; Table 3). A significant time × group interaction, indicating significantly greater improvement in the EMDR group than in the comparison group, was found for separation anxiety (F = 4.81, *p* = 0.033, partial η^2^ = 0.088), depression (F = 14.12, *p* < 0.001, partial η^2^ = 0.220), total anxiety (F = 6.95, *p* = 0.011, partial η^2^ = 0.122), and total anxiety-plus-depression (F = 6.41, *p* = 0.015, partial η^2^ = 0.114). The time × group interaction did not reach statistical significance for generalized anxiety (F = 3.20, *p* = 0.080), panic (F = 3.51, *p* = 0.067), social phobia (F = 2.57, *p* = 0.115), or obsessive–compulsive symptoms (F = 0.03, *p* = 0.861), indicating that these subscales improved over time in both groups without a significant difference in the magnitude of change (Table 3).

### 3.3. Between-Group Comparison and Post Hoc Pairwise Analyses

A significant main effect of group (i.e., a difference between groups collapsed across time) was found for generalized anxiety (F = 7.80, *p* = 0.007), obsessive–compulsive symptoms (F = 7.81, *p* = 0.007), depression (F = 11.40, *p* = 0.001), total anxiety (F = 7.56, *p* = 0.008), and total anxiety-plus-depression (F = 7.47, *p* = 0.009), reflecting generally higher scores in the comparison group across both timepoints; the group effect did not reach significance for panic (F = 3.73, *p* = 0.059) or social phobia (F = 1.42, *p* = 0.239). The corresponding between-subjects group-effect statistic for separation anxiety was not available in the analysis output used for this study; this gap is acknowledged in Section 4. Fisher’s LSD post hoc comparisons confirmed that pre-intervention scores did not differ significantly between the EMDR and comparison groups for any subscale except obsessive–compulsive symptoms (Section 3.1). At post-intervention, EMDR-group scores were significantly lower than the comparison group’s post-intervention scores for separation anxiety, generalized anxiety, panic, depression, total anxiety, and total anxiety-plus-depression (all *p* ≤ 0.033), and were also significantly lower than the comparison group’s own pre-intervention scores for most subscales. Within the comparison group, pre-to-post reductions reached statistical significance for separation anxiety (*p* = 0.028), generalized anxiety (*p* < 0.001), panic (*p* = 0.007), social phobia (*p* = 0.013), and obsessive–compulsive symptoms (*p* = 0.001) and total anxiety (*p* = 0.014), but not for depression (*p* = 0.130) or total anxiety-plus-depression (*p* = 0.247), whereas within the EMDR group, pre-to-post reductions were significant for all eight subscales (all *p* ≤ 0.002) (Table 3).

### 3.4. Glycemic Parameters

Mean blood glucose decreased significantly over the observation period in the EMDR group (221.1 ± 49.1 to 157.7 ± 36.3 mg/dL) but did not change significantly in the comparison group (199.7 ± 41.1 to 202.4 ± 47.0 mg/dL; Fisher’s LSD, *p* = 0.719). Two-way repeated-measures ANOVA showed a significant main effect of time (F = 34.21, *p* < 0.001) and, notably, a significant time × group interaction (F = 40.45, *p* < 0.001, partial η^2^ = 0.447), indicating a substantially greater reduction in blood glucose in the EMDR group; the main effect of group was not significant (F = 1.14, *p* = 0.291). A similar pattern was observed for mean daily insulin injection frequency, which decreased significantly in the EMDR group (3.27 ± 0.53 to 2.62 ± 0.50 injections/day) but did not change significantly in the comparison group (3.15 ± 0.46 to 3.35 ± 0.56 injections/day; *p* = 0.163): the time × group interaction was significant (F = 19.45, *p* < 0.001, partial η^2^ = 0.280), as was the main effect of group (F = 8.44, *p* = 0.005) (Table 4).

### 3.5. Adverse Childhood Experiences

The most frequently endorsed adverse life-event domains in this chart-based checklist were a family member’s serious illness (17.3%), witnessing a crime or accident (13.5%), exposure to violence or maltreatment (11.5%), increased family conflict (11.5%), and death of a family member (11.5%); all other domains were endorsed by fewer than 8% of participants. A total score on this checklist and its correlation with baseline psychological and glycemic measures were not computed for this study; only the domain-level endorsement rates reported above are available.

Among the EMDR group, the bilateral stimulation modality used was visual (eye movement) in 9 patients, tactile in 9 patients, and auditory in 7 patients (data missing for 1 patient).

## 4. Discussion

In this retrospective comparative study of children and adolescents with T1DM and comorbid anxiety/depressive symptoms, there was a significant main effect of time—an overall improvement averaged across both groups—for all eight RCADS anxiety and depression domains over the observation period (all *p* < 0.001). As detailed below, within-group comparisons showed that this improvement was significant in the EMDR group for every subscale, whereas the comparison group did not improve significantly on depression or total anxiety-plus-depression. Two-way repeated-measures ANOVA further showed that the EMDR group improved significantly more than the comparison group—that is, showed a significant time × group interaction—specifically for separation anxiety, depressive symptoms, total anxiety, and total anxiety-plus-depression, while improvements in generalized anxiety, panic, social phobia, and obsessive–compulsive symptoms did not differ significantly by treatment group. These findings are broadly consistent with the wider literature supporting EMDR as an effective intervention for anxiety and trauma-related symptoms [7,8], and extend this evidence to a pediatric population managing a demanding chronic illness.

Both groups experienced significant improvement in most anxiety domains over the observation period, which is not unexpected: routine psychiatric follow-up, including clinical support and, where indicated, pharmacotherapy, is itself an active intervention rather than a true no-treatment control. Notably, however, Fisher’s LSD post hoc comparisons indicated that the comparison group’s depressive symptoms and total anxiety-plus-depression scores did not improve significantly over the observation period (*p* = 0.130 and *p* = 0.247, respectively), whereas the EMDR group improved significantly on every subscale (all *p* ≤ 0.002); this pattern, together with the significant time × group interactions for separation anxiety, depression, total anxiety, and total anxiety-plus-depression, provides more direct statistical evidence of a differential EMDR-associated effect than a comparison of post-intervention scores alone would allow.

Contrary to our initial expectation of no differential metabolic benefit, mean blood glucose showed a significant time × group interaction favoring the EMDR group, decreasing significantly in the EMDR group but not changing significantly in the comparison group. A parallel pattern was observed for insulin injection frequency—the actual number of daily insulin administrations recorded, as distinct from the prescribed regimen—which likewise decreased significantly only in the EMDR group; because this measure can reflect adherence rather than glycemic status, it is interpreted here as a secondary, treatment-related finding rather than direct evidence of improved glycemic control (Section 2.3). Several non-exclusive explanations for the blood-glucose finding should be considered. First, reduced anxiety and depressive symptoms may have translated into improved diabetes self-management behaviors (e.g., more consistent CGM sensor use, insulin administration, and adherence to dietary and activity recommendations) specifically in the EMDR group. Second, although patients with a change in prescribed T1DM treatment regimen during the observation period were not included in the study (Section 2.1), day-to-day adherence could still have differed between groups in ways not captured by our data. Third, given the retrospective, non-randomized design and the absence of HbA1c or time-in-range data alongside the CGM-derived average glucose value used here (Section 2.3), this finding should be interpreted cautiously and confirmed in a prospective study with more granular glycemic outcome measures before concluding that EMDR has a direct metabolic benefit.

Cumulative adverse life events, ascertained via a thirteen-domain chart-based checklist, were relatively uncommon in this clinically referred sample; the most frequently endorsed domains were a family member’s serious illness, witnessing a crime or accident, exposure to violence or maltreatment, increased family conflict, and death of a family member. A total score on this checklist and its correlation with psychological or glycemic outcomes were not computed for this study, so it cannot be determined whether cumulative adverse life events were associated with baseline psychological or metabolic status in this sample; this remains an open question for future work using the broader ACE framework [5,6].

A baseline imbalance was observed for obsessive–compulsive symptoms, which were significantly higher in the comparison group than in the EMDR group; a corresponding significant main effect of group (collapsed across time) was also observed for generalized anxiety, obsessive–compulsive symptoms, depression, total anxiety, and total anxiety-plus-depression, indicating that the comparison group scored higher on these measures throughout the study period regardless of time. The proportion of male participants was higher in the EMDR group than in the comparison group, though this difference did not reach statistical significance (*p* = 0.095). Because treatment allocation was based on clinical decision-making rather than randomization, these patterns may reflect referral or selection tendencies (e.g., patients with more prominent obsessive–compulsive or generalized anxiety symptoms being preferentially directed to standard follow-up) rather than a random distribution of baseline severity, and should be considered when interpreting the between-group findings.

### Strengths and Limitations

This study is, to our knowledge, among the first to examine EMDR therapy specifically in children and adolescents with T1DM, jointly assessing psychological and glycemic outcomes alongside cumulative early-life adversity, using a two-way repeated-measures ANOVA that directly tests the time × group interaction rather than relying solely on post-intervention between-group comparisons. Several limitations should be acknowledged. First, the retrospective, non-randomized design precludes causal inference. Treatment allocation reflected clinical decision-making rather than a pre-specified, symptom-based algorithm, and although most baseline sociodemographic and clinical characteristics did not differ significantly between groups, residual confounding by clinical indication—that is, unmeasured factors that influenced both the decision to offer EMDR and the outcomes themselves—cannot be excluded. Second, although the two groups were of equal size (n = 26 each), the overall sample (N = 52) remains modest, particularly for detecting smaller time × group interaction effects such as those observed for generalized anxiety, panic, and social phobia. Third, RCADS and glycemic data were collected as part of routine clinical practice rather than a standardized research protocol; the number of valid CGM readings contributing to the three-month average glucose value, and whether CGM sensor use and reading availability were fully comparable between groups, were not verifiable from the centralized data source used (Section 2.3). Fourth, the study lacked a true no-treatment control group, limiting conclusions about the specific incremental efficacy of EMDR beyond standard psychiatric care; the typical frequency and content of routine follow-up visits, and the extent of pharmacotherapy exposure during the observation period, were not systematically documented in these retrospective records and could not be reported in detail. Fifth, single-center design and the absence of a longer-term follow-up period limit the generalizability and durability of the observed effects. Sixth, only the three-month average glucose value was available as a glycemic outcome; HbA1c and time-in-range data were not available, and this finding should be confirmed using more granular metrics in future prospective work. Insulin injection frequency was treated as a separate, secondary, treatment-related measure rather than as evidence of glycemic control per se, since it reflects the actual number of administered doses rather than a validated metabolic indicator (Section 2.3). Seventh, no outcome was designated as primary in the original study protocol; the eight RCADS subscales and the two glycemic parameters were therefore analyzed and are reported here as exploratory, without correction for the resulting number of unadjusted comparisons, and should be interpreted with corresponding caution. Eighth, effect sizes (partial η^2^) are reported in Table 3 and Table 4, but 95% confidence intervals could not be computed from the aggregated analysis output available for this study. Ninth, adverse life events were ascertained via a thirteen-domain chart-based checklist rather than a standardized, prospectively administered ACE questionnaire; chart-based ascertainment is likely to under-detect adverse experiences, no published psychometric reference for this checklist is cited here, and a total score together with its correlation with psychological or glycemic outcomes was not computed (Section 3.5). Tenth, although only patients who completed the planned course of EMDR sessions were included in the analysis, the number of patients (if any) who initiated but did not complete EMDR, and the reasons for non-completion, were not recorded in the data available for this study; if such patients existed and were systematically excluded, this could introduce additional selection bias favoring more engaged or less symptomatic patients in the EMDR group.

## 5. Conclusions

In this retrospective comparative study, EMDR therapy was associated with significantly greater improvement than standard psychiatric follow-up in separation anxiety, depressive symptoms, and overall anxiety burden (total anxiety and total anxiety-plus-depression scores), based on significant time × group interactions and within-group comparisons from two-way repeated-measures ANOVA. Unexpectedly, EMDR was also associated with a significantly greater reduction in mean blood glucose; a parallel pattern was observed for insulin injection frequency, a secondary, treatment-related measure. Neither blood glucose nor insulin injection frequency changed significantly in the comparison group. Cumulative adverse life events were relatively uncommon in this clinically referred sample, but their association with psychological or metabolic status could not be formally tested in this study. Because no outcome was pre-specified as primary and several sociodemographic and clinical details of treatment exposure could not be fully characterized from the available records, these findings should be considered exploratory and hypothesis-generating. They support further investigation of EMDR as an adjunctive psychological intervention in pediatric T1DM and highlight the need for prospective, adequately powered, randomized controlled trials with pre-specified primary outcomes, longer follow-up, and standardized metabolic outcome measures to confirm efficacy and clarify the relationship between EMDR, psychological symptoms, and glycemic control.

## Figures and Tables

**Table 1:** Sociodemographic and clinical characteristics by group.

Variable	EMDR (n = 26)	Comparison (n = 26)	*p*
Age, years (mean ± SD)	12.4 ± 2.6	12.9 ± 2.8	0.506
Sex, male, n (%)	15 (57.7)	9 (34.6)	0.095
Prior psychiatric admission, n (%)	7 (26.9)	2 (7.7)	0.140
Maternal psychiatric history, n (%)	4 (15.4)	4 (15.4)	1.000
Paternal psychiatric history, n (%)	1 (3.8)	0 (0.0)	1.000

SD: standard deviation. * *p* < 0.05 (chi-square test for categorical variables; independent-samples *t*-test for continuous variables).

**Table 2:** Baseline RCADS subscale scores by group.

RCADS Subscale	EMDR (n = 26)	Comparison (n = 26)	*p*
Separation anxiety	73.77 ± 8.92	74.77 ± 8.02	0.758
Generalized anxiety	53.35 ± 10.01	56.04 ± 6.97	0.283
Panic	66.69 ± 11.34	69.54 ± 11.42	0.446
Social phobia	54.92 ± 10.74	55.12 ± 8.69	0.949
Obsessive–compulsive symptoms	55.38 ± 12.76	62.92 ± 11.24	0.020 *
Depression	59.77 ± 11.84	64.08 ± 9.65	0.193
Total anxiety	63.15 ± 11.56	66.77 ± 12.05	0.333
Total anxiety + depression	63.23 ± 12.16	65.04 ± 14.78	0.641

Values are mean ± SD (RCADS T-scores; population mean = 50, SD = 10). Between-group baseline comparison: Fisher’s LSD post hoc test following two-way repeated-measures ANOVA. * *p* < 0.05.

**Table 3:** Pre- and post-intervention RCADS subscale scores within and between groups.

Subscale	EMDR Pre→Post	Comparison Pre→Post	Time × Group Interaction	Group Effect (Collapsed Across Time)
Separation anxiety	73.77 ± 8.92→59.15 ± 12.15	74.77 ± 8.02→68.62 ± 15.98	F = 4.81, *p* = 0.033 *, η^2^ = 0.088	Not available in analysis output
Generalized anxiety	53.35 ± 10.01→40.19 ± 7.33	56.04 ± 6.97→48.42 ± 11.00	F = 3.20, *p* = 0.080, η^2^ = 0.060	F = 7.80, *p* = 0.007 **
Panic	66.69 ± 11.34→52.65 ± 13.56	69.54 ± 11.42→62.31 ± 16.54	F = 3.51, *p* = 0.067, η^2^ = 0.066	F = 3.73, *p* = 0.059
Social phobia	54.92 ± 10.74→43.23 ± 10.62	55.12 ± 8.69→48.88 ± 12.66	F = 2.57, *p* = 0.115, η^2^ = 0.049	F = 1.42, *p* = 0.239
Obsessive–compulsive symptoms	55.38 ± 12.76→46.62 ± 8.05	62.92 ± 11.24→53.50 ± 13.12	F = 0.03, *p* = 0.861, η^2^ = 0.001	F = 7.81, *p* = 0.007 **
Depression	59.77 ± 11.84→45.38 ± 13.98	64.08 ± 9.65→60.85 ± 11.47	F = 14.12, *p* < 0.001 ***, η^2^ = 0.220	F = 11.40, *p* = 0.001 **
Total anxiety	63.15 ± 11.56→45.96 ± 12.82	66.77 ± 12.05→59.77 ± 16.52	F = 6.95, *p* = 0.011 *, η^2^ = 0.122	F = 7.56, *p* = 0.008 **
Total anxiety + depression	63.23 ± 12.16→46.54 ± 12.35	65.04 ± 14.78→60.92 ± 16.08	F = 6.41, *p* = 0.015 *, η^2^ = 0.114	F = 7.47, *p* = 0.009 **

F, *p*, and partial η^2^ for the time × group interaction and the main effect of group are from two-way repeated-measures ANOVA (df = 1.50 for all subscales unless noted). Within-group pre→post comparisons use Fisher’s LSD post hoc test. * *p* < 0.05, ** *p* < 0.01, *** *p* < 0.001.

**Table 4:** Glycemic parameters before and after the intervention period.

Parameter	EMDR Pre→Post	Comparison Pre→Post	Time × Group Interaction	Group Effect (Collapsed Across Time)
Mean blood glucose, mg/dL	221.1 ± 49.1→157.7 ± 36.3	199.7 ± 41.1→202.4 ± 47.0	F = 40.45, *p* < 0.001, η^2^ = 0.447	F = 1.14, *p* = 0.291
Insulin injections/day	3.27 ± 0.53→2.62 ± 0.50	3.15 ± 0.46→3.35 ± 0.56	F = 19.45, *p* < 0.001, η^2^ = 0.280	F = 8.44, *p* = 0.005

F, *p*, and partial η^2^ for the time × group interaction and the main effect of group are from two-way repeated-measures ANOVA (df = 1.50). Within-group pre→post comparisons use Fisher’s LSD post hoc test.

## Data Availability

The anonymized dataset supporting the findings of this study is available from the corresponding author upon reasonable request, since the data for this study were collected from a public institution with ethics committee approval.

## References

[1] de Wit M., Gajewska K.A., Goethals E.R., McDarby V., Zhao X., Hapunda G., Delamater A.M., DiMeglio L.A. (2022). ISPAD Clinical Practice Consensus Guidelines 2022: Psychological care of children, adolescents and young adults with diabetes. Pediatr. Diabetes.

[2] Reynolds K.A., Helgeson V.S. (2011). Children with diabetes compared to peers: Depressed? Distressed? A meta-analytic review. Ann. Behav. Med..

[3] Hilliard M.E., Yi-Frazier J.P., Hessler D., Butler A.M., Anderson B.J., Jaser S. (2016). Stress and A1c among people with diabetes across the lifespan. Curr. Diab. Rep..

[4] Buchberger B., Huppertz H., Krabbe L., Lux B., Mattivi J.T., Siafarikas A. (2016). Symptoms of depression and anxiety in youth with type 1 diabetes: A systematic review and meta-analysis. Psychoneuroendocrinology.

[5] Felitti V.J., Anda R.F., Nordenberg D., Williamson D.F., Spitz A.M., Edwards V., Koss M.P., Marks J.S. (1998). Relationship of childhood abuse and household dysfunction to many of the leading causes of death in adults: The Adverse Childhood Experiences (ACE) Study. Am. J. Prev. Med..

[6] Danese A., McEwen B.S. (2012). Adverse childhood experiences, allostasis, allostatic load, and age-related disease. Physiol. Behav..

[7] Shapiro F. (2018). Eye Movement Desensitization and Reprocessing (EMDR) Therapy: Basic Principles, Protocols, and Procedures.

[8] de Roos C., Greenwald R., den Hollander-Gijsman M., Noorthoorn E., van Buuren S., de Jongh A. (2011). A randomised comparison of cognitive behavioural therapy (CBT) and eye movement desensitisation and reprocessing (EMDR) in disaster-exposed children. Eur. J. Psychotraumatol..

[9] Christou A.I., Kalfadeli G., Bacopoulou F. (2026). Developmental Foundations of Psychosocial Interventions in Pediatric Oncology: A Lifespan Framework for Resilience. Children.

[10] von Elm E., Altman D.G., Egger M., Pocock S.J., Gøtzsche P.C., Vandenbroucke J.P., STROBE Initiative (2007). The Strengthening the Reporting of Observational Studies in Epidemiology (STROBE) statement: Guidelines for reporting observational studies. Lancet.

[11] Chorpita B.F., Yim L., Moffitt C., Umemoto L.A., Francis S.E. (2000). Assessment of symptoms of DSM-IV anxiety and depression in children: A revised child anxiety and depression scale. Behav. Res. Ther..

[12] Gormez V., Kılınçaslan A., Orengul A.C., Ebesutani C., Kaya İ., Ceri V., Nasıroğlu S., Filiz M., Chorpita B.F. (2017). Psychometric properties of the Turkish version of the Revised Child Anxiety and Depression Scale—Child Version in a clinical sample. Psychiatry Clin. Psychopharmacol..

[13] World Medical Association (2013). World Medical Association Declaration of Helsinki: Ethical principles for medical research involving human subjects. JAMA.

